# Experiences and support needs of older carers: A focus group study of perceptions from the voluntary and statutory sectors

**DOI:** 10.1016/j.maturitas.2019.02.003

**Published:** 2019-05

**Authors:** Nan Greenwood, Carole Pound, Raymond Smith, Sally Brearley

**Affiliations:** a Faculty of Health, Social Care and Education, Kingston University and St George’s, University of London, 6th Floor Hunter Wing, St George’s, University of London, London, SW17 0RE, United Kingdom; b Bournemouth University, Faculty of Health and Social Sciences, Royal London House, Lansdowne campus, Bournemouth University, Bournemouth, BH1 3LT, United Kingdom

**Keywords:** Informal carers, Older people, Experiences, Voluntary sector, Focus groups

## Abstract

**Objectives:**

Older informal carers play a vital, growing role in supporting others with long-term health conditions but their support needs and experiences are poorly understood. The aim of this study was to explore the perceptions of volunteers and professionals of the experiences and support needs of older carers (aged 70+ years).

**Methods:**

Thirty-five volunteers and professionals working with older carers in the voluntary and statutory sectors participated in a series of focus groups in outer London, United Kingdom. Groups were audio recorded, transcribed and subjected to thematic analysis.

**Findings:**

Five main themes were identified. These included participants’ perceptions of older carers’ ambivalence about asking for support, their multiple losses, often restricted lives, social isolation and loneliness and concerns for their loved ones when they can no longer care. Overall, these themes are similar to those reported for adult carers in general but older carers’ experiences were regarded as more challenging primarily because of their pride, attitudes to caring and because of their age, their own health was often declining making the physical aspects of caring and leaving their homes more difficult. Concerns about the future are thought to be particularly important for older carers of adult children with disabilities because they expect to be outlived by their children, although similar concerns were voiced by spouses of partners living with dementia.

**Conclusions:**

Professionals and volunteers need to consider these additional challenges for older carers. Support with accessing services, for example from the voluntary sector, is important as is future exploration of how to support older carers in planning for the future care for their loved ones is needed.

## Highlights

• This paper adds to what is known about the support needs of older carers.
• Older carers’ experiences may be particularly challenging.
• These carers’ ambivalence about requesting support may compound their difficulties.
• Loneliness and anxiety about their loved ones’ future care were highlighted.
• Their specific support needs require recognition by those working with older carers.

## 1 Introduction

Adult informal carers play a vital role in supporting others, often family members, with long-term health conditions. With ageing populations, their role is increasing. For example, in the United Kingdom (UK) carer numbers are expected to rise from an approximate 5.6 million currently [1] to 9 million in 2037 [2].

Being an informal carer has long been recognised as challenging although more recently the satisfactions of caring have been highlighted [3,4]. The reported negative effects of caring are numerous and include poor quality of life and emotional and physical problems of their own as a result of caring [5]. Evidence is also growing that not only does caring influence sleep but also physiological health markers [6].

Carers therefore deserve support not only for themselves but also because breakdowns in caring relations can be associated with poor outcomes for care recipients [7] and premature entry into residential care [8,9]. A range of interventions have been developed to support carers including psychoeducational, respite, counselling and more general support. Reviews of the effectiveness of these interventions lead to mixed results, but for example in carers of people with dementia, the evidence suggests that some interventions, especially psychoeducational and those with more than one component, may have the most positive impact [[10], [11], [12]].

Overall, numbers of carers are growing but numbers of older age groups (e.g. aged 65+) are increasing particularly rapidly. The total number of carers has risen by approximately 11% since 2001 but over the same period numbers of older carers rose by over three times this (35%). Amongst older carers, those aged 85 or older increased by 128% over the last decade [2].

However, despite the growing significance of older carers, research focussing on their experiences and support needs is limited [13,14]. This is both surprising and of concern for several reasons. For example, evidence suggests that older carers often provide more intensive support and care for longer hours [15] and they are also increasingly caring for someone with dementia [16] which is frequently thought to be one of the most challenging caring roles [17]. Greenwood and Smith [13] reviewed available literature relating to the experiences of older carers and identified little research specifically focussing on this group and suggested that without this, it was not possible to confidently say whether their experiences were any more or less challenging than other adult carers. Similarly, we do not know what the perceptions of those working with this group are for example, in terms of what they regard as older carers’ major challenges and perceptions of their needs. This is important to help understand where support is needed.

## 2 Aim

The aim of the study was to explore what volunteers and professionals working with unpaid older carers (70+ years) in the voluntary and statutory sectors understand to be the experiences and needs of older carers.

## 3 Methods

This study was undertaken in parallel to a focus group study with older carers investigating their experiences as older carers aged 70 years and above. Details of the perceptions of older carers can be found elsewhere [18]. A qualitative approach using focus groups was selected to help explore in-depth not only what is important to participants in their own words but also why they hold their opinions [19]. Focus groups also provide opportunities for discussion amongst peers with whom they share a common frame of reference and allow discussion and challenge between them [20]. One-to-one interviews were considered and do have some advantages but focus groups were selected in order to allow these discussions to take place.

Participants were recruited by two third sector carer organisations, both based in outer London, UK. Participants were either volunteers or professionals currently or recently (in the last two years) working with older carers. Given the fact that many people are delaying retirement to after 65 years, we decided to ask participants to focus their discussions on their perceptions of carers aged 70 years or older.

Sampling was purposive with the aim of recruiting a diverse range of volunteer and professional participants working in the voluntary and statutory sectors in terms of their roles and responsibilities. Recruitment was undertaken by two voluntary sector organisations who support carers in their geographical areas. This approach was selected because they had the knowledge and contacts needed to ensure a wide range of participants could be contacted. They also knew which organisations were most likely to be working with older carers. The recruiters had details of the study and it aims and methods and sent out copies of the participant information sheets to potential participants allowing them to make informed decisions about whether they wanted to participate. All participants had several days to decide whether they wanted to take part and it was emphasised that they were under no obligation to do so. Potential participants were then provided with details of focus group dates and locations.

All focus groups took place in the recruiting voluntary sector organisations and were digitally recorded and transcribed. Groups were facilitated by a researcher experienced in focus group facilitation. After giving written consent, participants were asked to provide some written background information including, for example, how long they had been working with older carers.

Facilitators used a brief topic guide. Areas covered included, for example, what participants thought older carers found challenging or satisfying about their role and what their support needs were.

Analysis was thematic [21] and ongoing during data collection making it possible to include ideas from earlier groups. Two authors read and re-read the transcripts ensuring familiarity and immersion in the data. The analysis process was iterative and started with independent open coding by the two authors focussing on two transcripts followed by discussion about the initial codes identified and emergent themes. Using these discussions between the authors as a basis, a preliminary coding framework was developed. Following this all the transcripts were analysed. Finally, the other authors made any final comments on the main themes to ensure that from their perspectives the main themes had been identified and described. Ethics approval was gained from the Faculty Research Ethics Committee (Ref: FREC 2017-11-004).

## 4 Findings

A total of 35 participants from the voluntary and statutory sectors (e.g. local authority, health and social care) participated in four focus groups. Most were female (89%) and were from the voluntary sector (86%). On average they had been working or volunteering with adult carers for over eight years and specifically with older carers for slightly less time. Participants were in a variety of roles including: volunteer befrienders, support workers and commissioning managers. Overall the groups had similar profiles in terms of their ages although on average, the participants from the statutory sector had been working with carers in general and older carers specifically for longer.

Table 1 provides details of the participant demographics.

**Table 1:** Participant demographics.

	Voluntary sector	Statutory sector	Total n=35
	30 (86%)	5 (14%)	35 (100%)
**Gender**			
Female	27 (77.1)	4 (11.4)	31 (89%)
Male	3 (8.6)	1 (2.9)	4 (11%)
**Age** (years)			
Mean	51.9	53.6	52.1
Median	52.5	50	52
Range	26-75	43-69	26-75
**Time working with carers (years)**			
Mean	7.1	18.4	8.7
Median	5	20	5
Range	1–30	2–30	1–30
**Time working with older carers (years)**			
Mean	5.7	14	6.9
Median	3	14	3
Range	<1–20	2–26	<1–26

### 4.1 Themes

The themes identified during analysis are described in detail below. They included older carers’ ambivalence about asking for support, their many losses, often restricted lives, social isolation and loneliness, concerns about when they can no longer care, caring satisfactions and support needs. Anonymised quotes are provided. F represents female and M male participants.

#### 4.1.1 Ambivalence about asking for support

Evidence suggests that adult carers in general often fail to access available support. However, participants here thought that older carers may be even less likely to request support because of their attitudes and expectations regarding their role.

‘*I would say the experiences are different, they’re different because younger carers are more likely to seek support, to probably get some help for the people they’re caring for, whereas my experience with the older carers, so they just get on with it*.’ (F12)

Sometimes this was because they saw it as a natural role, integral to who they were, for example, if they were caring for an adult child with learning or physical disabilities. One participant commented that older carers often say:

‘*…it’s my nature, it’s what I’ve always done, I just get on with it, and it’s I’ve been doing this all my life, so it just comes out naturally*.’(F13)

They were also thought to be ambivalent about asking others for help, as older carers often believed only they provided the best care.

‘*Nobody can do it as well as I can sort of thing, yeah, I think*.’ (F20)

Embarrassment at admitting they were unable to cope or unwillingness to reveal personal information here also play a part.

‘*It’s there’s an embarrassment in part from the carer admitting that you’ve got an issue still, and a tiredness because you’ve had it for so long*.’ (M17)

Possibly because many older carers had lived through post-war austerity, the phrase ‘make do and mend’ was often cited.

‘*A lot of our older carers do sort of just get on with it and do, make the best of life, and perhaps don’t access the right support because of that, because they’re just getting on with it*.’(F1)

Participants reported that the older carers they worked with often said they did not want to ‘bother’ their children. They said a frequent comment was:

‘*I don’t want to trouble her (daughter), she lives far away, she’s got her own issues*.’(F21)

Guilt and feeling a burden themselves for asking for support were also highlighted.

‘*One thing that you get with older carers that you don’t get anywhere near as much with younger adults, is they feel like a burden themselves, and there’s a lot of guilt around asking for help*.’ (F6)

It was also thought that older carers found it difficult to have help coming in their homes because older care recipients are often very resistant to accepting help especially if this meant ‘strangers’ coming into their homes. This had an impact on carers.

‘*Also, I think maybe the, obviously they're of a different generation, but they’ve not had… they don’t like strangers coming into their environment, they’d rather look at having their wife wash them or whatever*...’ (F22)

#### 4.1.2 Multiple losses

As with younger adult carers, being an older carer was thought to include loss in many facets of their lives. This includes loss of life outside caring, loss of an anticipated shared future and lost or changed relationships.

‘*…loss of the person that they were with, or planned a future with, and that is a big thing because when you grow up, you retire, you think I actually wanted to go travelling with my husband and now he’s got dementia and I’m looking after him and I have lost the husband*.’ (F24)

This volunteer who had been a carer herself for many years talked about her sense of loss when her child was diagnosed with psychotic illness but reflected that this also applied to those caring for someone with dementia.

‘*…coming to terms with loss, you’re lost, you’re losing the person and it will happen with dementia as well, you’re losing the person that you had hopes for*.’ (F2)

Participants were also aware that being a carer was associated with loss of companionship and being able to enjoy social activities or going on holiday together.

‘…*they feel that they’ve lost something, especially when there’s dementia involved as well, like I’ve had carers say to me,“We used to go dancing all the time and we can’t anymore*.”’ (F7)

Especially if carers have been caring for a long time, loss of the caring role and the challenges of creating a life after caring were highlighted.

‘*I think it’s harder for an older, or much harder for an older carer to resume a life once they lose their caring role, because probably they’ve lost some of their friends as well along the way through age related issues*.’ (F23)

#### 4.1.3 Being housebound, carers’ own poorer health and having restricted lives

Many older carers that participants worked with spend most of their time at home alone with their loved ones. There are a wide variety of reasons for this including carers’ own health affecting, for example, mobility and care recipients’ health conditions. As older people themselves, these carers find physically helping care recipients especially challenging.

Older carers’ own declining health was seen as adding to their difficulties.

‘*I think there’s a little bit more that needs to be done for older carers, much more than just respite, sometimes they have health conditions themselves*.’ (F1)

They also have fewer people to whom they can turn for help.

‘*They often have their own disabilities or illness as they get older, it makes the situation far worse, and have fewer friends or family around, friends who die, family who die, so they are becoming more and more on their own and less supported*.’ (F14)

Conditions such as dementia or autism may not only make it physically difficult to leave home but carers are also sensitive to others’ reactions and may therefore avoid going out.

‘… *I think what puts people off social activities and anything from just local groups or to trips out, is the thought that it would be so much more difficult to manage this person away from home*.’ (F14)

Caring for someone with stigmatised conditions, such as severe mental illness or dementia, were regarded as having a considerable impact on socialising.

‘*It’s very important because carers and people with schizophrenia are really isolated because they don’t, they can’t talk about it with their next-door neighbour, because they immediately think that she’s living next to someone who’s going to, you know, do a hatchet job*.’ (F26)

#### 4.1.4 Loneliness and social isolation

The participants stressed that loneliness and isolation were particularly common amongst older carers. This was partly, but not solely, due to the fact that they were more likely to be housebound. It was also recognised that many older carers were isolated because many of their friends may have died or were no longer able to visit.

‘… *her health is not good and she’s now having to look after her husband who’s starting with dementia and she sometimes goes weeks without seeing anybody …. I think after a time if you keep saying, “Well sorry I can’t go out*,” … *people just dwindle, and in the end, you can count on one hand the number of people that maybe would still support you*.’ (F7)

However, there is also often loneliness within the relationship especially when caring for someone with dementia.

‘*You’ve got a situation where they’re struggling much more to cope, physically and emotionally, and the person that they’re wanting to provide that love and care for isn’t the person as such, I know the body is the same, but in terms of the character and personality is separate, it’s not the same person*.’ (M4)

#### 4.1.5 Concerns about the future when they can no longer care for their loved ones

A common issue highlighted by these participants was older carers’ anxiety about the future care of their loved ones when they died or could no longer care. This was seen to be particularly worrying for carers of adult children with disabilities but also applied to some spouses caring for partners with conditions such as dementia.

‘*They talk a lot about this Yes…. after I have gone… what happens, after I have gone? It’s a huge, huge worry*.’ (M18)

One participant said a carer had even admitted to her that she hoped her son would die first.

‘*I’m very sorry to tell you this but I want him to die before me,’ and I find that very difficult because I never think like that*.’ (F21)

Worrying about the costs of care was a significant aspect of this.

‘… *they try and put things in place in terms of trying to sort things out with the financial side, trusts, whatever it may be, if they’ve got funds on that side that’s appropriate, or they try and get this person maybe some access to social care so that there’s some kind of contact with agencies before this person can no longer care for them*.’ (M17)

However, despite being a cause of anxiety, many carers were thought to try and avoid confronting this until a crisis.

‘*So what will happen if something happens to…?’ ‘I don’t want to think about that, if that happens I’ll deal with it then,’ ‘But then you’re at crisis point, are you going to be able to think clearly about what you need, about what your wife needs? … but it’s getting through to somebody who is older that actually they do need to get something in place*.’ (F15)

Participants recognised that other family members may not want to talk about it either.

‘*I’m looking at learning disabilities and obviously there’s a lot of carers who are getting older and older that have grown children at home and they don’t want to talk about future planning*.’ (F16)

## 5 Discussion

Many of the participants here had worked for a considerable time with both older and younger adult carers making them ideally placed to make direct comparisons with older and younger adult carers’ perceived experiences and needs. Overall being an older carer was seen by these volunteers and professionals as particularly hard for a wide range of reasons. Perhaps most importantly older carers’ apparent belief that they should not need to ask for help and the common resistance of the care recipients to outside support, was perceived to reduce the likelihood they would seek support.

Many of the issues for older carers described by the participants have been highlighted before for adult carers in general. Loneliness and social isolation are commonly reported amongst carers [1,22] but according to our participants these challenges are generally worse for older carers. They thought that the fact that carers themselves are ageing means they are often less mobile and also find the physical challenges of caring and taking their loved ones out even more difficult. Supporting someone with stigmatised conditions such as dementia was also thought to make socialising with the care recipient harder which is part of the reason carers often enjoy services intended for both carers and their loved ones [23]. Carers’ concerns for how their loved one will be supported once they can no longer care has also been reported before but generally in relation to carers of adult children with disabilities [24,25] rather than carers of spouses with dementia. This deserves further investigation.

There are many similarities with the findings from these focus groups with professionals and volunteers with the themes identified in the focus groups with older carers (70+ years) themselves [18]. Themes describing issues such as loneliness, isolation and anxiety about planning for when they can no longer care were common to both groups. However, the study with carers suggests they paid greater attention to the challenges of caring that were exacerbated by their own ageing and reduced energy and others' unwillingness to talk about when they die. Although this was highlighted by the volunteers and professionals here, it was not given nearly as much emphasis. The issue of talking about and planning for the future was also raised by the older carers themselves but the volunteers and professionals here appeared to think that the main barrier to planning for the future was the carers themselves who were often unwilling to confront it until there was a crisis.

These different perceptions of the experiences of older carers have implications for supporting this important group. It may be that volunteers and professionals underestimate the impact of age on older carers and on their ability to care. The fact that these participants emphasised older carers’ perceived resistance to seeking assistance may also mean that those working with them are less persistent in offering support. These different perspectives need to be explored further.

The study has several strengths. The participants had a wealth of experience working closely with older carers in a range of diverse roles. The focus groups were lively with extensive discussion between participants and they clearly showed where they agreed or disagreed with each other. Importantly, there was a lot of similarity in themes across the four focus groups suggesting that they were relevant across these geographical areas.

However, there are limitations. The participants here worked in outer London boroughs which may not be typical of volunteers and professionals in other geographical areas. Also, although the group facilitators tried to ensure that the participants focused their discussion on older carers, at times it was unclear if they were referring to all adult carers or only the older age groups. At analysis, where there was uncertainty about this, the data were not used. Future research might consider using one-to-one interviews to make it easier to control this. However, this would lose some of the advantages of focus groups.

## 6 Conclusions

Our findings suggest that according to these participants from the voluntary and statutory sectors there are a number of practical steps that can be taken for supporting older carers. This age group are believed to often need greater support than younger carers but may be less likely to admit to this or to be able to find out about services. Older carers are also thought to perhaps be less persistent in their efforts to access support both because of their ambivalence about asking for help but also because of their reducing energy levels. This suggests that those providing support to older carers may need to be more pro-active in their approach. More thought needs to be given to providing services aimed at both the carer and the care recipient – they often want to be together and many feel unwilling to have services coming into their homes. Finally, these people working with older carers believe more help needs to be given to older carers to support them with talking about and planning for the support needs of their loved ones when they can no longer care through death or illness.

## Contributors

Nan Greenwood conceived of and led the study, undertook data analysis and drafted the paper.

Raymond Smith also undertook the data analysis.

All authors collected the data and approved the final version of the paper.

## Conflict of interest

The authors declare that they have no conflict of interest.

## Funding

The Wellcome Trust funded the study [209343/Z/17/Z]. The Trust was not involved in the study in other respects.

## Ethical approval

Ethical approval was granted by the Faculty of Health, Social Care and Education, Kingston University research ethics committee (Ref FREC 2017-11-004). Participants consented to anonymised direct quotes being presented.

## Provenance and peer review

This article has undergone peer review.

## Research data (data sharing and collaboration)

There are no linked research data sets for this paper. The data are confidential.
